# Supplementary material for: Development of a new high-yield integration site assay reveals disease-specific patterns across HTLV-1-associated pathologies
Source: Microbiol Spectr. 2025 Mar 20;13(5):e03208-24. doi: 10.1128/spectrum.03208-24 (PMC12054188; doi:10.1128/spectrum.03208-24)
Supplement: Fig. S1 — Results of an IS experiment on a 1% agarose gel. [file spectrum.03208-24-s0001.docx]

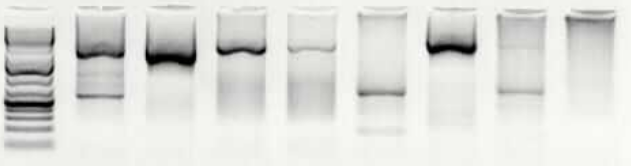


**Supplementary Figure 1:** Results of an integration site experiment on a 1% Agarose gel. Positive wells have an amplicon band around 1.5-5 kb, while negative results can present either as a smear or a 500pb band. Because the primer used for Sanger sequencing does not overlap with the others, neither the 500pb amplicon nor the smear can produce false-positive integration site sequences using Sanger sequencing.

5000

1500

500

Molecular Weight

Positive

Positive

Positive

Positive

Negative

Positive

Negative

Negative
